# Deep Learning Strategy for Sliding ECG Analysis during Cardiopulmonary Resuscitation: Influence of the Hands-Off Time on Accuracy

**DOI:** 10.3390/s23094500

**Published:** 2023-05-05

**Authors:** Vessela Krasteva, Jean-Philippe Didon, Sarah Ménétré, Irena Jekova

**Affiliations:** 1 Institute of Biophysics and Biomedical Engineering, Bulgarian Academy of Sciences, Acad. G. Bonchev Str. Bl 105, 1113 Sofia, Bulgaria; vessika@biomed.bas.bg; 2 Schiller Médical, 4 Rue Louis Pasteur, 67160 Wissembourg, France; jean-philippe.didon@schiller.fr (J.-P.D.); sarah.menetre@schiller.fr (S.M.)

**Keywords:** deep neural networks, CNN, ECG, arrhythmia, CPR, VF, shockable rhythm, continuous ECG analysis, out-of-hospital cardiac arrest

## Abstract

This study aims to present a novel deep learning algorithm for a sliding shock advisory decision during cardiopulmonary resuscitation (CPR) and its performance evaluation as a function of the cumulative hands-off time. We retrospectively used 13,570 CPR episodes from out-of-hospital cardiac arrest (OHCA) interventions reviewed in a period of interest from 30 s before to 10 s after regular analysis of automated external defibrillators (AEDs). Three convolutional neural networks (CNNs) with raw ECG input (duration of 5, 10, and 15 s) were applied for the shock advisory decision during CPR in 26 sequential analyses shifted by 1 s. The start and stop of chest compressions (CC) can occur at arbitrary times in sequential slides; therefore, the sliding hands-off time (sHOT) quantifies the cumulative CC-free portion of the analyzed ECG. An independent test with CPR episodes in 393 ventricular fibrillations (VF), 177 normal sinus rhythms (NSR), 1848 other non-shockable rhythms (ONR), and 3979 asystoles (ASYS) showed a substantial improvement of VF sensitivity when increasing the analysis duration from 5 s to 10 s. Specificity was not dependent on the ECG analysis duration. The 10 s CNN model presented the best performance: 92–94.4% (VF), 92.2–94% (ASYS), 96–97% (ONR), and 98.2–99.5% (NSR) for sliding decision times during CPR; 98–99% (VF), 98.2–99.8% (ASYS), 98.8–99.1 (ONR), and 100% (NSR) for sliding decision times after end of CPR. We identified the importance of sHOT as a reliable predictor of performance, accounting for the minimal sHOT interval of 2–3 s that provides a reliable rhythm detection satisfying the American Heart Association (AHA) standards for AED rhythm analysis. The presented technology for sliding shock advisory decision during CPR achieved substantial performance improvement in short hands-off periods (>2 s), such as insufflations or pre-shock pauses. The performance was competitive despite 1–2.8% point lower ASYS detection during CPR than the standard requirement (95%) for non-noisy ECG signals. The presented deep learning strategy is a basis for improved CPR practices involving both continuous CC and CC with insufflations, associated with minimal CC interruptions for reconfirmation of non-shockable rhythms (minimum hands-off time) and early treatment of VF (minimal pre-shock pauses).

## 1. Introduction

Cardiac arrest is a life-threatening state of circulatory failure due to a loss of cardiac systolic function. It is the result of four specific cardiac rhythm disturbances: ventricular fibrillation (VF), pulseless ventricular tachycardia (VT), asystole, and pulseless electrical activity [1]. Cardiac arrest remains a significant cause of morbidity and mortality worldwide [2]. Recommendations on the quality-of-life support state that cardiopulmonary resuscitation (CPR) should be delivered to patients in cardiac arrest, and it should be combined with electrical shock if VF or VT is present [3]. High-quality CPR includes minimal chest compression (CC) pauses defined as a pre-shock pause < 10 s in the case of manual defibrillation and CC fraction > 60% in the case of continuous CC. These goals are dedicated to Advanced Life Support of cardiac arrest and are based on evidence for improved resuscitation outcomes in patients with shockable rhythms [4,5] and increased return of spontaneous circulation (ROSC) with shorter peri-shock pauses [6,7].

In out-of-hospital cardiac arrest (OHCA), both CPR and an automated external defibrillator (AED) should be used according to a strict Basic Life Support algorithm in which CPR periods are interrupted every 2 min for regular heart rhythm analysis by the AED [8]. The resulting shock advisory decision is taken automatically by digital signal processing algorithms using the electrocardiogram (ECG) in a single defi-lead acquired between the two defibrillator pads placed on the chest. The quality of the ECG signal affects the shock advisory performance; therefore, the regular AED analysis on a clean ECG is performed when the AED gives verbal (and/or visual) instructions to rescuers not to touch the victim. Outside these short hands-off time slots, the ECG signal during CPR is commonly distorted by strong CC artefacts [9]. Although CCs are provided at a quasi-periodic rate (100–120 min^−1^) [8], their impact on the defi-lead ECG is quite variable with respect to waveform patterns and frequency content, mostly related to the combination of the delivery of CC, the patient, and the rescuer. An ECG rhythm blurred by CC artefacts can hardly be determined visually by experts, but can be automatically determined by the shock-advisory algorithms implemented in AEDs. Existing approaches for CC artefact suppression mostly rely on adaptive filters that use external reference signals from sensors for impedance or compression depth [10,11,12,13,14,15,16,17,18], compression force [19], compression acceleration [20], arterial blood pressure [21], etc. Several approaches seeking a simpler AED implementation use only one ECG input, where periodic CC artefacts are suppressed by pattern matching algorithms [22], coherent line removal [23], and Kalman filters [24]. Short-time Fourier transform images of the ECG spectrum have been shown to be effective for filtering out CC artefacts while processed by a condition-based filtering algorithm [25] and deep convolutional encoder/decoder [26]. Whether with or without reference signals, the aforementioned methods have common disabilities in providing filtered ECG signals either with insufficiently suppressed CC artefact components or distorted ECG waves. These warped ECG signals limit the accuracy of the automated shock advisory algorithms whether they are conventionally trained for ECG signals without artefacts [13,15,16,17,19,20,25,26] or apply specially optimized decision rules for detection of ventricular fibrillation during CPR [22,27].

Currently, solutions for rhythm analysis during CPR implemented in AEDs follow two strategies:

1. Two-stage algorithms are implemented in the real-time AED analysis process during OHCA interventions, applying the first stage during uninterrupted CC (analysis duration 11–30 s), eventually followed by a second reconfirmation stage on clean ECG (5–9 s). A delayed shock decision with reconfirmation analysis is required in 26–100% of OHCA interventions analyzed by several commercial AED algorithms [28,29,30,31]. Such two-stage schemes demand synchronization with additional algorithms for detection of the start and stop of CC in a standard CPR protocol with compression-to-ventilation ratios of 30:2, 15:2, or 15:1 [32,33,34].
2. Single-stage algorithms based on deep neural networks (DNN) are run in PC workstations with OHCA databases during CPR. The DNN input feature maps and architectures depend on study-specific processing concepts, e.g., supplying unfiltered raw ECG signals with continuous CC artefacts to the input of fully convolutional neural networks (CNNs) [35], prefiltered raw ECG signals to CNN [36,37], or a hybrid DNN architecture, including a combination of convolutional layers, residual blocks, and bidirectional long short-term memory (LSTM) layers [38].

Neither the two-stage algorithms nor DNN models trained on ECG during uninterrupted CC can benefit from analyzing the rhythm during the short insufflation periods, even though they are the unique timeslots with clean ECG samples that are commonly used by experts for visual determination of the rhythm during CPR [39]. Although there are shock advisory technologies with reliable performance for short analysis intervals (3–10 s) on clean ECG [34,39,40,41], there are certain limitations that restrict the real-life application of this technology during insufflations in OHCA. These limitations are mainly related to uncontrollable factors, concerning the ECG signal quantity (indefinite or very short duration of insufflations, quartile range 2.5–11.4 s [39,40]) and quality (presence of movement artefacts, unreliable localization using ECG, and/or impedance signal). When also considering that the rhythm may spontaneously convert from non-shockable to shockable (refibrillation) or vice versa (ROSC or conversion of a shockable rhythm to asystole) at any time during the OHCA resuscitation procedure [42,43], continuous rhythm monitoring during CPR is important. Providing early treatment of fibrillation (minimal pre-shock pauses) or maintaining uninterrupted CPR for asystole and organized rhythms (minimum hands-off time) is of particular benefit to patient outcome [44]. A few studies have investigated the strategy for continuous ECG processing during 2 min of uninterrupted CPR, showing that continuous ECG filtering [45] or rhythm analysis of sequential ECG clips [46] can reduce the frequency of useless CPR interruptions. Although these were retrospective studies that have not been clinically implemented, they suggested that rhythm analysis output could be used to guide resuscitation in real time.

This study aims to investigate the potential of CNN as an end-to-end feature extraction and classification algorithm that can continuously provide a shock advisory decision during CPR without preselection conditions for the presence and absence of CC during analysis. A deep learning strategy by sliding ECG analysis (analysis durations of 5, 10, and 15 s) of a large number of OHCA interventions during CPR has been shown feasible for training a CNN model, which is able to self-extract valuable ECG rhythm information from short CC interruptions. Thus, a substantial improvement of the shock advisory performance can be achieved during hands-off periods, whereby a hands-off time of slightly above 2 s is sufficient for the CNN to satisfy the American Heart Association (AHA) standards for AED rhythm analysis [47]. With competitive performance during CPR, the presented deep learning strategy is a basis for improved CPR practices with minimal CC interruptions for rhythm reconfirmation, thereby providing minimized hands-off intervals for non-shockable rhythms, and minimized pre-shock pauses for early treatment of ventricular fibrillations.

## 2. Materials and Methods

### 2.1. ECG Databases

The study used a proprietary clinical ECG database (Schiller Medical SA, Wissembourg, France) provided for research purposes and for the retrospective investigation of OHCA rhythms during CPR. It consisted of ECG and thoracic impedance records from commercial AEDs (DEFIGARD TOUCH 7, Schiller Medical SA, France) used during OHCA interventions by the Paris Fire Brigade (BSPP, Brigade des Sapeurs-Pompiers de Paris) in the period January–December 2017. The reanimation protocol applied CPR with a 30:2 compression-to-ventilation ratio and CC rate of 100–120 min^−1^, paused every 2 min for a regular AED rhythm analysis, following the European Research Council (ERC) Adult Basic Life Support guidelines [48]. The database was anonymized before the study to ensure the medical confidentiality, without information about the patient identity, epidemiological data, diagnosis, drug therapy, or outcome.

**Figure 1:**
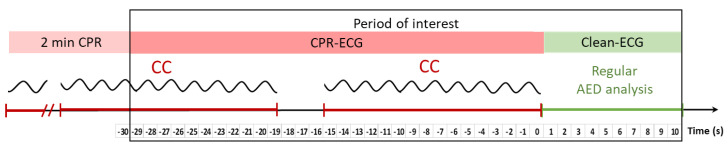
Illustration of the period of interest in OHCA interventions.

The period of interest in OHCA interventions was defined after at least 2 min of CPR, starting from 30 s before to 10 s after the begin of the regular AED analysis at 0 s, as illustrated in Figure 1. The period of interest is further denoted as the time interval (−30 s; 10 s]. Thus, the reference moment at 0 s splits two sequential ECG buffers within the period of interest:

- CPR-ECG: 30 s ECG buffer (−30 s; 0 s] contaminated by CC episodes and hands-off pauses during insufflations. Notably, the instants for start and stop of CC episodes can occur randomly during the period of interest.
- Clean-ECG: 10 s ECG buffer (0 s; 10 s] with hands-off pause and presumably without artefacts, representing the ECG signal analyzed by the AED real-time process.

Accordingly, Clean-ECG was used for visual revision of the rhythm by three emergency physicians with experience in cardiac arrest cardiology from BSPP. The rhythm annotation followed the AHA classification scheme [47]:

- VF: coarse ventricular fibrillation with amplitude > 200 µV;
- NSR: normal sinus rhythm with visible P-QRS-T waves and heart rate of 40–100 bpm;
- ONR: other non-shockable rhythm, including atrial fibrillation/flutter, sinus bradycardia, supraventricular tachycardia, premature ventricular contractions, heart blocks, etc.;
- ASYS: asystole with low-amplitude ECG, having peak-to-peak signal deflection ≤ 100 µV for more than 4 s.

All cases with lack of consensus between annotators or noise during regular AED analysis were excluded from this study. Following the AHA statement [47], the annotations were grouped into a shockable Sh (VF) and a non-shockable NSh (NSR, ONR, and ASYS) category. An additional screening process was managed to select only cases with a consistent shock advisory decision throughout the 40 s period of interest, given that the annotations covered rhythm observations during the last 10 s. All cases with transitions from Sh to NSh (e.g., ROSC) or from NSh to Sh (e.g., refibrillation) were considered inconsistent and excluded. Since transitions between different NSh rhythms did not lead to a change of the shock advisory decision, they were considered consistent. Rhythm consistency was validated automatically for all periods of interest, which are disclosed between two sequential regular AED analyses with NSh decision, or between successful AED shock and sequential regular AED analysis with NSh decision, or between unsuccessful AED shock and sequential regular AED analysis with Sh decision. All other cases underwent manual revision of the ECG rhythm in the CC pause for insufflations just preceding the period of interest. The rhythm during this CC pause was compared to the ground-truth annotation at the end of the period of interest to confirm consistency (Sh → Sh, NSh → NSh). 

As detailed in Table 1, we used an OHCA database from 2838 patients, with a total number of 13,570 extracted periods of interest, including annotations for 802 VF, 352 NSR, 3824 ONR, and 8592 ASYS. The database was split patient-wise into two uniform parts for independent learning (1504 patients and 7173 periods of interest) and testing (1334 patients and 6397 periods of interest). Relative rhythm distributions were similar for learning and testing, distinctive to the real-life proportion of OHCA rhythms: most cases present with ASYS (about 63%) and ONR (about 28%), and rare cases with VF (about 6%), NSR (about 3%). 

### 2.2. Sliding ECG Analysis during CPR

**Figure 2:**
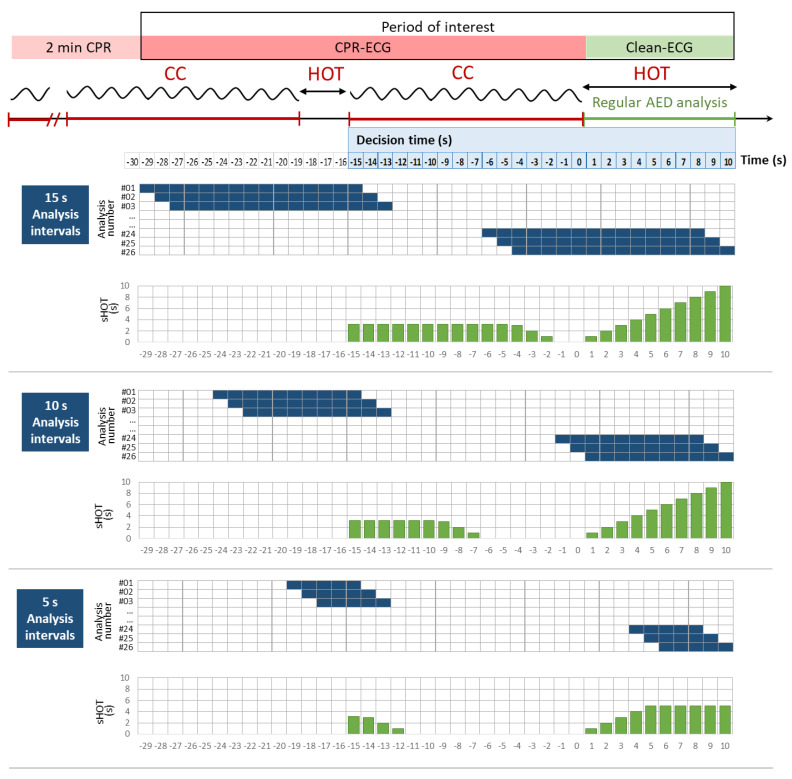
Strategy for continuous ECG analysis during CPR with 26 decision times [−15 s; 10 s] in the period of interest by sliding analysis intervals with durations of 15 s, 10 s, and 5 s (blue horizontal bars). Trend of computed sliding HOT (sHOT) over the 26 decision times (green vertical bars). CC: chest compressions; HOT: hands-off time; CPR-ECG: ECG during CPR; Clean-ECG: ECG during regular AED analysis.

The period of interest was continuously analyzed by the shock-advisory system in sliding intervals shifted by 1 s (Figure 2). Three analysis intervals were defined (5 s, 10 s, and 15 s) to investigate the effect of different ECG signal durations on performance, as these were commonly used in published CPR analysis schemes [28,29,31,46]. The decision times of all analysis intervals were aligned in the range [−15 s; 10 s], given that the decision was taken in the last second of analysis. Thus, a total number of 26 analyses shifted by 1 s were assigned within the period of interest, as illustrated in Figure 2.

We measured the hands-off time (HOT) as the duration of CC interruptions exceeding 1 s, e.g., due to insufflations or regular AED rhythm analysis (illustrated in Figure 2). CC interruptions in OHCA were detected during the real-time process of the AED interventions using the impedance wave analysis algorithm validated in [34]. Additionally, we decided it was important to measure the amount of HOT that represents the total duration of the clean ECG parts in each sliding analysis interval. Therefore, we define the parameter sliding HOT (sHOT) as the cumulative duration of CC interruptions over a sliding analysis interval. For sHOT computation, HOT episodes in each sliding analysis interval are not necessarily contiguous. The change of sHOT in sequential ECG analyses during CPR is illustrated in Figure 2, evaluated over analysis intervals of 5 s, 10 s, and 15 s.

### 2.3. DNN Design

**Figure 3:**
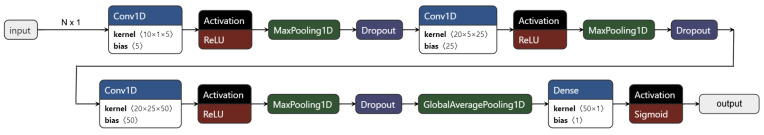
The CNN model architecture applied in this study for sliding ECG analysis during CPR. The model was visualized using the free online tool Netron [50].

The module for continuous ECG analysis during CPR is based on an end-to-end DNN that directly inputs ECG from defi-pads, extracts features in hidden layers, and outputs the shock advisory decision. Optimization of the DNN architecture is not the focus of this study, which rather investigates principles for deep learning with both uninterrupted (continuous) and partially interrupted CC during analysis, timed according to Figure 2. We assume that an optimized DNN architecture for the most complex shock advisory task during uninterrupted CC artefacts would also be feasible for scenarios with partially interrupted CC. Thus, the DNN design takes into account our previous optimization study for VF detection during continuous CC [35] by adopting the published best model with a fully convolutional architecture. The hyperparameters shown in Figure 3 were optimized in [35] by random search of 1500 CNN models with 2–7 convolutional layers, 5–50 filters, and 5–100 kernel sizes. Performance stability of the selected CNN model has been demonstrated for slow, normal, and rapid compressions, as well as for strong, moderate, and weak CC artefacts [35]. Another important consideration for choosing the fully convolutional design presented below is its relatively simple computational profile that is compatible with the computational capabilities of embedded AED systems with real-life clinical use. A proof of effective CNN implementation in the hardware of an AED was demonstrated in [49], wherein a five-layer CNN was embedded for the regular shock advisory decision on clean ECGs.

The CNN design in Figure 3 takes an input from a single defi-lead ECG (bandwidth 1–30 Hz, sampling rate 125 Hz). The size of the input feature vector is 1 channel × N, where N = 625, 1250, and 1875 denotes the number of ECG samples used for analysis durations of 5 s, 10 s, and 15 s, respectively. The CNN model consists of three convolutional blocks followed by a global average pooling (GlobalAveragePooling1D) layer and an output dense block. Each convolutional block (i = 1, 2, 3) includes the following:

- Conv1D: 1D convolution layer with kernel dimensions < K_i_ × F_i-1_ × F_i_ >and biases corresponding to F_i_, where the kernel sizes are K_1,2,3_ = {10, 20, 20}, and the number of filters is F_1,2,3_ = {5, 25, 50}. Consider F_0_ = 1, matching the input ECG dimension;
- Activation: activation layer applying rectified linear unit (ReLU) function;
- MaxPooling1D: max pooling layer with a pool size of 2;
- Dropout: dropout layer with a drop rate α = 0.3.

The role of the global average pooling layer is to downsample the output of each filter in the third convolutional block to a single average value. Thus, a smaller vector with one feature per filter (50 features) is supplied to the input of the final dense block. It includes a layer with fully connected neurons with 50 weights (*w_i_*) and one bias (*b*) followed by the final activation layer, implementing a sigmoid activation function for a binary classification. The latter outputs the probability for presence of shockable rhythm *pSh* ∈ [0; 1]:

(1) $$pSh(x)=\frac{1}{1+e^{-x}},\;\mathrm{where}\;x=\sum_{i=1}^{50}w_{i}{GlobalAveragePooling1D}_{i}+b.$$

### 2.4. DNN Training 

Three independent CNN models with analysis durations of 5 s, 10 s, and 15 s, namely, CNN-CPR (5 s), CNN-CPR (10 s), and CNN-CPR (15 s) respectively, were trained. The training followed the scheme for sliding analysis intervals in Figure 2, applying 26 sequential analyses with decision times [−15 s, −14 s, …+9 s, +10 s] within the period of interest of each intervention in the learning database. Thus, the number of signals in the learning database (Table 1) was multiplied by 26, resulting in a total number of 186,498 analysis intervals: 10,634 (VF), 4550 (NSR), 51,376 (ONR), and 119,938 (ASYS). The learning database was additionally partitioned to two training/validation subsets in a ratio 70/30%.

The CNN-CPR models were programmed in Python 3.9 using Keras with Tensorflow backend. The training was conducted on a workstation PERSY Stinger with an Intel CPU Xeon Silver 4214R@2.4 GHz with two processors (Intel Corporation, Santa Clara, CA, USA), 96 GB RAM, NVIDIA RTX A5000-24GB GPU.

The models were compiled with the following settings:

- Kernel initializer: ‘random uniform’;
- Optimizer: ‘Adam’ with learning rate of 0.001, and exponential decay rate for the first and second moment estimates β1 = 0.9 and β2 = 0.999, respectively;
- Loss function: ‘weighted binary cross-entropy’ for two target classes (Sh/NSh). Considering the unequal distribution of Sh (5.7%, 409/7172) and NSh signals (94.3%, 6763/7172) in the learning database, a penalty proportional to the class prevalence was applied in the loss (Equation (2)), where M is the size of the learning database, δ_m_ is a binary indicator function (δ_m_ = 1 if x_m_ belongs to the shockable class; otherwise, δ_m_ = 0), and w_Sh_ = 0.943 and w_NSh_ = 0.057 are the weights for Sh and NSh classes, complying with the condition w_Sh_+ w_NSh_ = 1.

(2) $$\mathrm{Loss}=-\frac{1}{M}\sum_{m}^{M}\delta_{m}w_{\mathrm{Sh}}\log\left(P\left(x_{m}\in Sh\right)\right)+\left(1-\delta_{m}\right)w_{NSh}\log\left(1-P\left(x_{m}\in Sh\right)\right).$$

The training process fitted the model with the following settings:

- Training epochs: maximum of 750, with activated early stopping if no improvement in the validation loss was obtained for 150 epochs;
- Batch size: 128.

Each of the models (CNN-CPR (5 s), CNN-CPR (10 s), and CNN-CPR (15 s)) was trained with five independent runs. The model with the minimum loss in the validation dataset among the five runs was subjected to further independent evaluation with the test dataset.

### 2.5. Performance Evaluation

The detection accuracy for Sh rhythms (VF) and NSh rhythms (NSR, ONR, and ASYS) was evaluated with the standard metrics for reporting the performance in AEDs [47] in terms of sensitivity (Se) and specificity (Sp):

(3) $$Se=\frac{TP}{TP+FN},\;Sp=\frac{TN}{TN+FP},$$

where true positive (TP) and true negative (TN) were the correctly detected Sh and NSh cases, respectively, while false positive (FP) counted the NSh classified as Sh, and false negative (FN) accumulated the Sh cases that were recognized as NSh. 

For the purpose of continuous ECG analysis, the mean values and 90% confidence intervals (CI) of the accuracy metrics (Se and Sp) in the test database were reported for 26 decision times in the interval [−15 s; 10 s] as defined in Figure 2. Parameter sHOT was also computed for the same decision times. Assuming a nonparametric distribution, sHOT median value, interquartiles, and min–max range were reported. Grouping of accuracy metrics (Se, Sp) in 12 sHOT intervals (0 s, (0–1 s], … (9–10 s], >10 s) was used to study the effect of CC interruptions on CNN-CPR model performance. The statistical analysis was conducted using the software Statistica 7 (Dell Inc., Round Rock, TX, USA).

## 3. Results

### 3.1. Validation Receiver Operating Characteristic Curve

**Figure 4:**
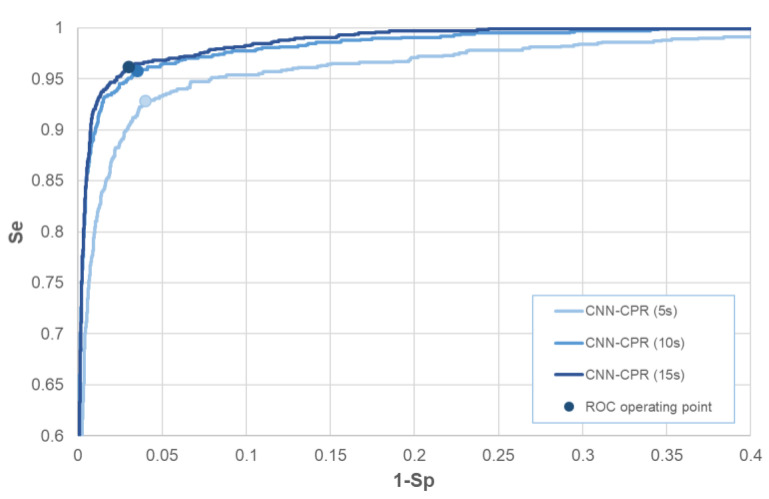
Validation ROC curves for rhythm analysis during CPR with models CNN-CPR (5 s), CNN-CPR (10 s), and CNN-CPR (15 s). The choice of the ROC operating points respects the condition Se + Sp→max.

At the end of training and selection of the minimum loss models CNN-CPR (5 s), CNN-CPR (10 s), and CNN-CPR (15 s), they were subjected to receiver operating characteristic (ROC) curve analysis with the validation database (Figure 4). The area under the ROC curve (ROC-AUC) was estimated to be 0.9813, 0.9921, and 0.9938, respectively. The main result of this analysis is the choice of the ROC operating point, i.e., the determination of the threshold applied to the output *pSh* (Equation (1)) that provides optimal ROC performance in the validation database. The optimization strategy applied to the ROC curve was maximization of the sum Se + Sp→max, which was shown to be trustworthy in previous studies with binary shock advisory decision [35,41]. The chosen operating points for the three models are highlighted in Figure 4. 

### 3.2. Sliding CPR Analysis: Case Study

**Figure 5:**
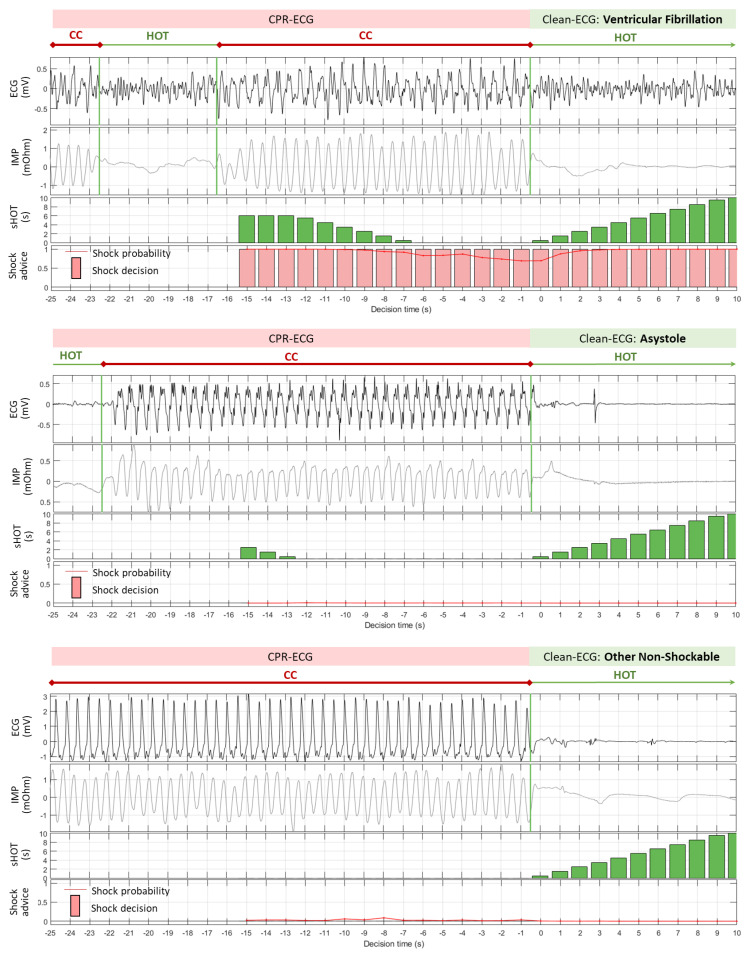
Examples of sliding rhythm analysis during CPR provided by model CNN-CPR (10 s): correct shock advice for all decision times while analyzing VF (**top**), ASYS (**middle**), and ONR (**bottom**). Two parameters, sHOT and shock advice (shock probability [0; 1] and shock decision (yes/no) applying shock probability threshold = 0.15), were computed for a 10 s ECG buffer in anteriority to the decision times [−15 s; 10 s] with a step of 1 s.

**Figure 6:**
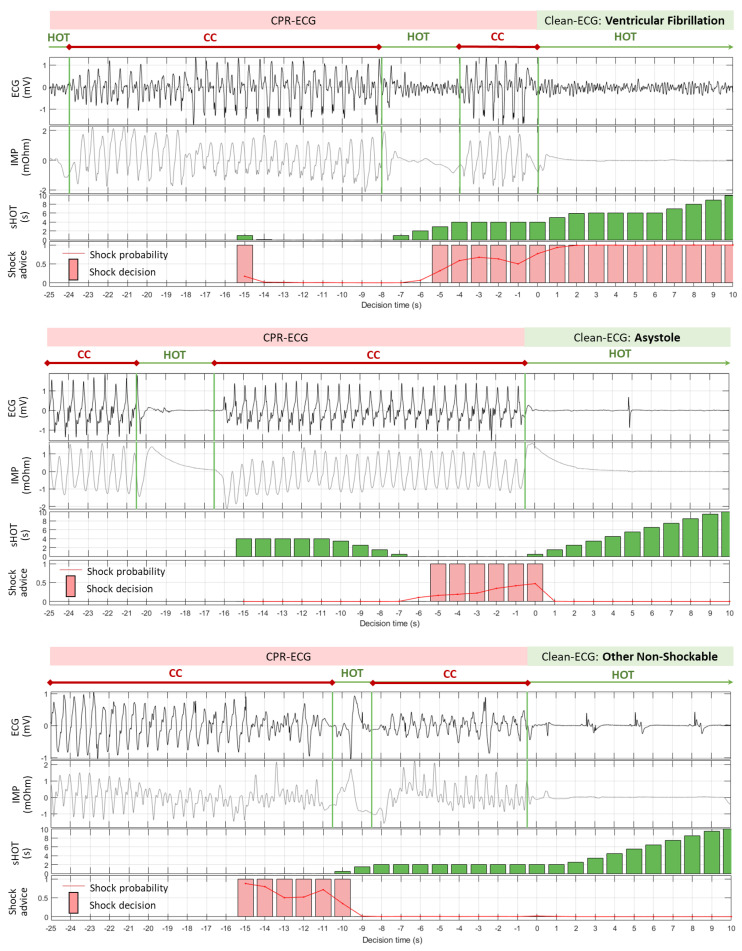
Examples of sliding rhythm analysis during CPR provided by model CNN-CPR (10 s): erroneous shock advice for some decision times while analyzing VF (**top**), ASYS (**middle**), and ONR (**bottom**). Two parameters, sHOT and shock advice (shock probability [0; 1] and shock decision (yes/no) applying shock probability threshold = 0.15), were computed for a 10 s ECG buffer in anteriority to the decision times [−15 s; 10 s] with a step of 1 s.

The six examples in Figure 5 and Figure 6 illustrate the principle of 1 s sliding ECG analysis during CPR with 26 decision times in the interval [−15 s; 10 s]. Different scenarios for CC-contaminated ECG rhythms (VF, ASYS, and ONR) were visualized by ECG and the additional impedance variation signal (IMP). The IMP signal was not included in the analysis but used only for the purpose of better illustration of the CC deflections and their interruptions during insufflations or regular AED analysis. The resulting sHOT and shock advice computed for each decision time of the model CNN-CPR (10 s) are shown in separate bar plots.

The first VF example (Figure 5, top) presents a correct shock advice for all decision times based on a very high probability for a shockable rhythm that is slightly influenced by the HOT during analysis: *pSh* ≥ 0.9 for sHOT > 1 s; *pSh =* 0.65–0.8 for sHOT ≤ 1 s. The CPR artefacts in the second VF example (Figure 6, top) resembled a QRS-like waveform that was challenging for the model CNN-CPR (10 s) in nine analysis steps [−14 s; −6 s] during continuous CC (sHOT = 0–2 s), giving erroneous NSh decisions based on a very low shock probability *pSh* < 0.05. The insufflation pause starting at −8 s triggered a correct Sh decision after −5 s, where the shock probability gradually increased as sHOT increased.

The correctly detected NSh rhythms, i.e., ASYS (Figure 5, middle) and ONR (Figure 5, bottom), showed almost zero *pSh* for all 26 decision times. These examples demonstrate the robust performance of the model CNN-CPR (10 s) for long continuous CC episodes (sHOT = 0 s), as well partially interrupted CC episodes (sHOT > 0 s). Nevertheless, analyses during sHOT = 0 s presented the most challenging conditions for rhythm detection, as illustrated in the two examples with erroneous Sh detection: (i) ASYS [−5 s; 0 s] in Figure 6 (middle); (ii) ONR [−15 s; −10 s] in Figure 6 (bottom). Just a little over 1 s of clean ECG (sHOT > 1 s) improved specificity by sharply reducing *pSh* to zero.

### 3.3. Sliding CPR Analysis: Statistical Study

**Figure 7:**
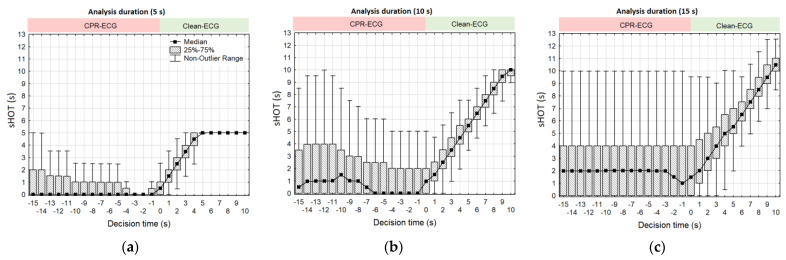
Statistical box plots of sHOT distribution over decision time estimated for the test OHCA database and analysis durations of 5 s (**a**), 10 s (**b**), and 15 s (**c**). The analysis interval covers a buffer with anteriority to the decision time [−15 s; 10 s], sliding in steps of 1 s.

**Figure 8:**
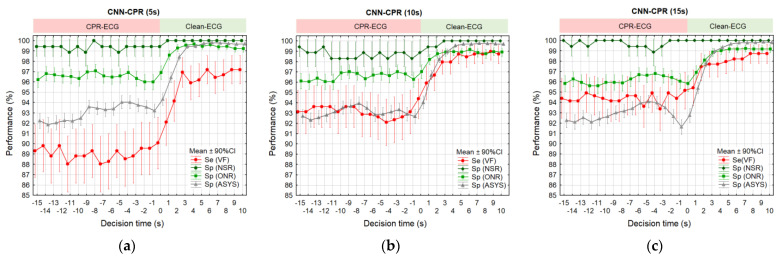
Statistical distributions of the test performance over decision time estimated for CNN-CPR models with analysis durations of 5 s (**a**), 10 s (**b**), and 15 s (**c**). The statistics are derived for predefined arrhythmia categories (VF, NSR, ONR, and ASYS) within decision time [−15 s; 10 s], sliding in steps of 1 s.

The proportion of CC artefacts varies in sliding CPR analysis and might strongly influence the rhythm performance. Therefore, sHOT as a measure of the cumulative analysis time without CC artefacts is an essential CPR characteristic that is further reported. In our retrospective study, sHOT was not supervised by human annotators or by machine rhythm analysis algorithms, but was a rather uncontrollable factor depending on the recorded real-life CPR scenarios in our OHCA database. As shown in Figure 7, sHOT could vary from zero to the CNN analysis duration, reaching a maximum of 5 s, 10 s, or 15 s for models CNN-CPR (5 s), CNN-CPR (10 s), CNN-CPR (15 s), respectively. Figure 7 focuses on the statistical distributions of sHOT over time in the test database, which is further synchronized with the test performance over time in Figure 8, evaluated by a sliding plot every 1 s.

Figure 7 shows a relatively stable sHOT distribution along the decision time [−15 s; 0 s], followed by a linear increase in sHOT over the Clean-ECG period [1 s; 10 s]. During the CPR-ECG period [−15 s; 0 s], CNN-CPR models presented test performance in the following ranges:

- CNN-CPR (5 s): mean Se (VF) = 88–90%, Sp (ASYS) = 91.5–94%, Sp (ONR) = 96–97%, Sp (NSR) = 99–100% (Figure 8a).
- CNN-CPR (10 s): mean Se (VF) = 92–94.4%, Sp (ASYS) = 92.2–94%, Sp (ONR) = 96–97%, Sp (NSR) = 98.2–99.5% (Figure 8b).
- CNN-CPR (15 s): mean Se (VF) = 93.4–95%, Sp (ASYS) = 91.5–94%, Sp (ONR) = 95.6–96.8%, Sp (NSR) = 99–100% (Figure 8c).

Performances increased right after CC stopped during Clean-ECG period [1 s; 10 s]. It took approximately 3 s to reach the maximum performances that spanned the following ranges:

- CNN-CPR (5 s): mean Se (VF) = 96–97.2%, Sp (ASYS) = 99.4–99.8%, Sp (ONR) = 99.2–99.6%, Sp (NSR) = 100% (Figure 8a).
- CNN-CPR (10 s): mean Se (VF) = 98–99%, Sp (ASYS) = 98.2–99.8%, Sp (ONR) = 98.8–99.1%, Sp (NSR) = 100% (Figure 8b).
- CNN-CPR (15 s): mean Se (VF) = 97.5–98.8%, Sp (ASYS) = 97.8–99.8%, Sp (ONR) = 98.1–99.2%, Sp (NSR) = 100% (Figure 8c).

### 3.4. Test Performance vs. sHOT

**Figure 9:**
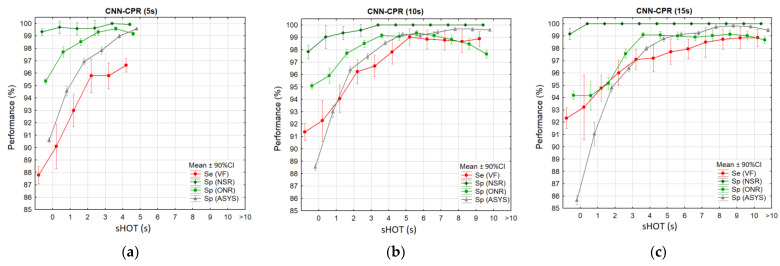
Statistical distributions of the test performance vs. sHOT estimated for CNN-CPR models with analysis durations of 5 s (**a**), 10 s (**b**), and 15 s (**c**). The statistics are derived for predefined arrhythmia categories (VF, NSR, ONR, and ASYS) in 12 sHOT intervals: 0 s, (0–1 s], …, (9–10 s], >10 s.

Investigating the concept for sliding rhythm analysis during CPR, it is relevant to study all sequential analysis decisions together. Therefore, we defined a common decision set of all analyses in the period of interest, including 26 sliding analyses with four performance metrics per analysis (Se (VF), Sp (ASYS), Sp (ONR), and Sp (NSR)), which were described over time in Figure 7. This common decision set was studied in groups quantified by sHOT, thus identifying the subset with continuous CC (sHOT = 0 s) or other subsets partially or fully interrupted with CC (sHOT > 0 s). The performance results in 12 sHOT intervals (0 s, (0–1 s], …, (9–10 s], >10 s) are shown in Figure 9 (mean value ± 90% CI) and Table 2 (mean value) for the three CNN-CPR models with analysis durations of 5 s, 10 s, and 15 s. All models presented the lowest performance during continuous CC (sHOT = 0 s), which monotonically improved as sHOT increased in the analysis interval (sHOT > 0 s). Limitations have been noted for some models due to their inability to satisfy the AHA performance goals [47] for some rhythms during continuous CC, i.e., model CNN-CPR (5 s) with Se (VF) = 87.8%, Sp (ASYS) = 90.6%; CNN-CPR (10 s) with Sp (NSR) = 97.8%; CNN-CPR (15 s) with Sp (ONR) = 94.2%, Sp (ASYS) = 85.7%. An important result that can be read from Figure 8 and Table 2 is the minimal sHOT interval, which benefited rhythm analysis to achieve the following performance above the AHA goals:

- Minimal sHOT = (0–1 s] was necessary for the model CNN-CPR (5 s) to improve Se (VF) to 90.1%, and CNN-CPR (10 s) to improve Sp (NSR) to 99%;
- Minimal sHOT = (1–2 s] was necessary for the models CNN-CPR (5 s) and CNN-CPR (10 s) to improve Sp (ASYS) to 96.9% and 96.4%, respectively, and CNN-CPR (15 s) to improve Sp (ONR) to 95.2%;
- Minimal sHOT = (2–3 s] was necessary for the model CNN-CPR (15 s) to improve Sp (ASYS) to 96.4%.

## 4. Discussion

This study presented a novel deep learning algorithm for sliding shock advisory decision during CPR, managing both CPR practices with continuous CC and CC with insufflations. To the best of our knowledge, this is one of the first studies to evaluate Sh and NSh rhythm detection performance as a function of the hands-off-time during analysis. The results in Figure 9 and Table 2 present a certain scientific novelty, suggesting the importance of sHOT as a reliable predictor of performance, confirmed for three analysis durations (5 s, 10 s, and 15 s). In particular, the scenario with continuous CC (sHOT = 0 s) had the worst performance, which favorably increased monotonically as sHOT increased. We accounted for sHOT intervals as low as 2 s that could provide a reliable rhythm detection satisfying the AHA performance goals for AEDs. Such a duration of hands-off pauses during insufflations occurs on a regular basis in CPR protocols with CC to insufflations ratios of 30:2 and 15:2. Therefore, the developed algorithm for sliding CPR analysis can take real advantage of clean ECG episodes during insufflations to improve the shock advisory performance related to both NSh rhythms with minimized CC interruptions for rhythm reconfirmation and Sh rhythms with early CC interruption for shock treatment. Such ideas for rhythm analysis during insufflation pauses were previously presented by Ayala et al. [39] and Ruiz et al. [40] as a strategy for reliable diagnosis before the end of 2 min CPR cycles. These studies identified CC pauses with duration (2.5–11.4 s interquartile range) occurring nearly every 20 s in a 30:2 CPR scenario. Approximately 89–95% of CC pauses which were identified either by manual [40] or thoracic impedance analysis [39] were long enough to launch a conventional AED shock advice algorithm for pauses > 3.5 s. Instead, our study provides CNN-CPR models that can continuously monitor the ECG rhythm during CPR without dealing with detection issues and waiting for usable CC pauses. Even when there are no insufflations in CPR protocols with continuous CC, the presented algorithm can take a reliable shock advisory decision using shorter pre-shock pauses than conventional AED analysis algorithms. The presented sliding CPR analysis technology with primary binary rhythm classification and supplementary *pSh* probability can give regular feedback to the rescuers during CPR. However, the options for application of this technology were not disclosed in this paper. This implies the need for additional investigation on the optimal practices that can lead to improved clinical effect.

The key characteristic of the developed CPR analysis technology is the use of deep CNN with direct input of raw ECG in a single lead setting. Beneficially, there is no need for prefiltering, additional measurements of time–frequency ECG features, or additional reference inputs. In the presented sliding strategy for rhythm monitoring without preselection conditions for the presence and absence of CC, the application is greatly simplified because the rhythm analysis is not triggered by an external algorithm for detection of start and stop of CC. This breakthrough is made possible by the novel training strategy, involving ECG signals during CPR in 26 sequential analyses shifted by 1 s within the period of interest. Considering the intra- and inter-rescuer variations of the CC rate, number of CCs, and duration of insufflations, the training analysis intervals take place at arbitrary moments during the CPR periods. The inclusion of hands-off periods with varying length during regular AED analysis have also helped the deep learning process. It is worth noting that such a strategy for training of DNN models is efficient by using sufficiently large real-life OHCA interventions available in this study. Although the training dataset collects several sequential analyses for one intervention, the test process shows the independent performance of each sequential analysis, without interactions with other previous analyses. Thus, the results in Figure 8 are representative of Se/Sp measurements at different moments during the period of interest, taking one observation for each intervention. The test for robustness to the uncontrollable factor associated with sHOT variance, however, requires pooling all consecutive analyses across all interventions categorized by sHOT. Thus, the statistics in Figure 9 and Table 2 are representative of as many cases as possible for the sHOT variance in terms of duration and position within the analysis intervals and related transitions during CC/insufflations, which stop and start at arbitrary instants.

This study also compared three CNN-CPR models with different analysis durations (5 s, 10 s, and 15 s). The training process in Figure 4 based on the validation ROC curves showed substantial improvement in CNN-CPR performance from 5 s to 10 s, while the difference between 10 s and 15 s was negligible. The detailed analysis of the test performance over time in Figure 8 shows that model CNN-CPR (5 s) had a limited Se (VF) of 88–90% (CPR-ECG) and 96–97.2% (Clean-ECG). Favorably, Se (VF) improved to 92–95% (CPR-ECG) and 97.5–99% (Clean-ECG) for both longer analysis durations of 10 s and 15 s. From Figure 8, we can deduce that the specificities for all studied analysis durations (5 s, 10 s, and 15 s) were comparable in both parts of the period of interest, i.e., CPR-ECG (91.5–94% ASYS, 95.5–97% ONR, and 98.2–100% NSR) and Clean-ECG (99–99.8% ASYS, 99–99.5% ONR, and 100% NSR), identified in the plateau of maximum performance with decision times from 3 s to 10 s. 

**Figure 10:**
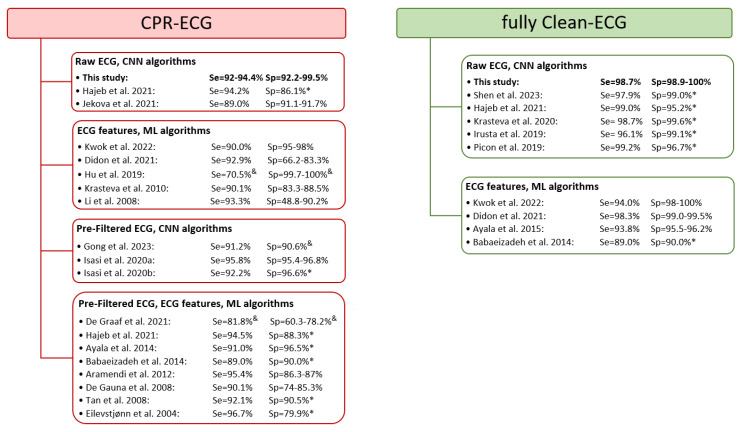
Comparison with state-of-the-art algorithms for analysis of CPR-ECG and fully Clean-ECG signals. This study reports (bolded font) the test performance for CNN-CPR (10 s) as the min–max range for Se (VF), Sp (NSR, ONR, and ASYS) with results taken at decision time in the range [−15 s; 0 s] for CPR-ECG, and decision time equal to 10 s for fully Clean-ECG. Other studies are included only if they reported performance for OHCA databases, cited as found in the original articles: Se (VF) and Sp (min–max range reported for rhythms NSR, ONR, and ASYS, where available). Sp * relates to specificity values that are applied to a mixed dataset (ONR, ASYS, and/or NSR) in published studies. Se, Sp ^&^ denotes studies which used an indefinite rhythm category that was neither true positive nor true negative in the context of a binary shock advisory decision. The studies referred in the figure are as follows: Hajeb et al., 2021 [38], Jekova et al., 2021 [35], Kwok et al., 2022 [46], Didon et al., 2021 [31], Hu et al., 2019 [29], Krasteva et al., 2010 [22], Li et al., 2008 [27], Gong et al., 2023 [52], Isasi et al., 2020a [36], Isasi et al., 2020b [37], De Graaf et al., 2021 [30], Hajeb et al., 2021 [25], Ayala et al., 2014 [14], Babaeizadeh et al., 2014 [17], Aramendi et al., 2012 [16], De Gauna et al., 2008 [24], Tan et al., 2008 [20], Eilevstjønn et al., 2004 [51], Shen et al., 2023 [49], Krasteva et al., 2020 [41], Irusta et al., 2019 [53], Picon et al., 2019 [54], Ayala et al., 2015 [39]. CNN: convolutional neural network; ML: machine learning.

The ECG analysis concept in this study brings a new perspective to the state of the art. The algorithm monitors the rhythm during continuous CPR, not limited to analysis only during CC [14,16,17,20,22,24,25,27,29,30,31,35,36,37,51,52] or only during clean ECG [17,31,39,41,49,53,54], as summarized in Figure 10. Although most published algorithms have been optimized and report the performance for either CPR-ECG or fully Clean-ECG signal parts during OHCA, the shock advisory performance of our algorithm CNN-CPR (10 s) is in the high range for both signal parts: CPR-ECG (Se = 92–94.4%, Sp = 92.2–99.5%) for decision time in the range [−15; 0 s]; fully Clean-ECG (Se = 98.7%, Sp = 98.9–100%) for decision time equal to 10 s. 

The difficult task for CPR-ECG analysis was managed here without additional processing (ECG prefiltering, and additional sensors), which can be found in some studies [14,16,17,20,36,37,51]. A major factor, which limits the performance of algorithms relying on CC filtering, is the overlap between the spectra of CC artefacts and the dominant VF and QRS components [15,55]. Common effects of prefiltering are suppressed VF and QRS amplitudes, as well as elevated CC artefact residuals that are reported to seriously disturb the asystole baseline detection [16,17,56,57]. The latter warrants attention because of the need to report an independent performance for the asystole group, given that this is the dominant rhythm in OHCA. The performance for asystole defined the lowest limit of the Sp range in this study (92.2%), and other studies (48–91%) that follow this straight report practice for CPR-ECG [16,22,24,27,29,30,31,35]. The highest outlier values for asystole Sp (about 95%) were recorded in [36,46], demonstrating that performance can be potentially improved by integrating information from previous analyses and an external accelerometer sensor. The additional use of CNN after precise prefiltering adjusted to the instantaneous CC frequency gives a benefit to the final shock advisory decision during CPR-ECG, as seen in the highest results reported by Isasi et al. [36], which comply with AHA goals [47] for all rhythms, including asystole (Sp > 95%). Our study also relies on the CNN technology without prefiltering, achieving one of the best performance results. We suggest that these results are due to the effective filtering of CC artefacts by deep hidden convolutional layers. In our previous study [35], we turned attention to the self-extracted features after convolutional filters, illustrating their relevance for the enhancement of ECG components specific to the rhythm and for the suppression of the corrupting CC artefacts. Properly trained CNN filters are a prerequisite for better results. Therefore, this study focused on enhanced CNN training in a deep learning strategy that takes advantage of sHOT variance through sliding ECG analysis, leading to improved Se (3–5.5% points) and Sp (1–8% points) compared to [35].

Only two recent studies were found to address a similar concept for continuous shock-advisory decisions during CPR managed by a single algorithm [38,46]. Hajeb et al. [38] presented the concept in an unrealistic scenario with artificially mixed Physionet ECG and OHCA asystoles with CC at different signal-to-noise ratios. Although the DNN design was more complex with CNN, residual, and LSTM layers, as well as an additional short-time Fourier transform of the raw ECG input, the performance Sp was limited to 86% (CPR-ECG) and 95% (Clean-ECG). Kwok et al. [46] presented real-life OHCA rhythms during CPR and a machine learning algorithm that analyzes continuous ECG sequences in 5 s clips through wavelets, hidden semi-Markov modeling, and random forest classification. Their shock advisory performance was adjusted to higher Sp than Se, such that Se was limited to 90% (CPR-ECG) and 94% (Clean-ECG). Both studies demonstrated different preferences for the balance between Se and Sp; nevertheless, strategies for their improvement should be sought. This study effectively applied a deep learning strategy with sliding ECG analysis. The trained CNN models are as good as previous ones optimized on Clean-ECG datasets [41], and superior to those previously trained by CPR-ECG datasets with noninterrupted CC [35]. The latter, associated with an improvement in Se by 3–5.5% points and Sp by 1–8% points, is potentially due to the effective use of hands-off pauses during CPR. 

Current technologies for rhythm analysis during CPR embedded in real AED devices require a priori knowledge of the presence or absence of CC. Information about the presence of CC is needed in order to compute an index of confidence to underscore reliable results [29,30] or to trigger a fast reconfirmation analysis while CCs are stopped [28,29,31]. Therefore, the performance of AED algorithms during CC with either reduced Se (70.5% [29] or 81.8% [30]) or reduced Sp (66.2–83.3% [31] or 60.3–78.2 [30]) should be considered in the context of their specific two-step rhythm analysis schemes, which rely on shock advice reconfirmation analysis in 26–100% of cases [31]. DNN technologies have the potential for improving current practices although they are mostly tested offline with databases in GPU-based development platforms. A recent study [49] took a further step toward embedding computationally efficient deep CNN models in the setting of a digitally connected defibrillator. A perspective for the near future would be that properly trained CNN architectures such as the one under investigation in this study offer optimal solutions that can improve current therapy. 

## 5. Limitations

Our database was composed of several ECG episodes during CPR from a single AED intervention as independent samples. This was applied given that single episodes were distanced by at least 2 min intervals in the electronic recording of the intervention, according to the hypothesis that the process driving the morphology of the CC artefact is nonstationary due to changes in the underlying ECG rhythm (shock delivery, drug injection, refibrillation, and return of spontaneous circulation), as well as in the delivery of CC over time (fatigue of the rescuer, swap of rescuers, etc.). 

Although our database was extracted from more than 2500 OHCA patients, the limitation regarding the absence of shockable ventricular tachycardias VTs (<0.15%, given a number of 18 rapid VTs from a total of 13,570 episodes) did not allow reporting the related performance results. This phenomenon may be partially related to the fixed data collection period (1 year), limiting the possibility to extend the collection of some rare rhythms, such as rapid VTs.

## 6. Conclusions

This study contributes to the enhanced application of neural networks for shock advisory decisions during CPR. The applied novel deep learning strategy for sliding ECG analysis in the presence and absence of CC achieves substantial performance improvement in short hands-off periods, such as insufflations or pre-shock pauses, even if CC resumes before the final CNN decision. The statistical study in Figure 9 demonstrates the importance of sHOT as a reliable predictor of performance (the longer the sHOT interval, the higher the performance of the algorithm). A minimum sHOT > 2 s was found to provide reliable rhythm detection, meeting the AHA standards for AED rhythm analysis [47], whatever the analysis period (5, 10, or 15 s). Although it could be anticipated that a longer hands-off time results in a more reliable shock advisory decision of any conventional algorithm designed to analyze clean ECGs, this cannot be foreseen for algorithms designed for specific measurements on ECGs with CC artefacts. Nevertheless, previous studies did not continuously estimate the drop in performance at the outset of CC. Additionally, our paper contributes to a quantification of the recovery in performance after CC artefacts have ceased.

The final results from this study shed light on the general application of CNN-CPR models by reporting their performance on a fully independent test database, which to our knowledge is one of the largest with real-life cardiac arrest rhythms during CPR. Our best model CNN-CPR (10 s) presented a combination of high Se and high Sp with CPR-ECG (92–94.4% for VF, 92.2–94% for ASYS, 96–97% for ONR, and 98.2–99.5% for NSR) and with Clean-ECG (98–99% for VF, 98.2–99.8% for ASYS, 98.8–99.1 for ONR, and 100% for NSR). The performance can be considered competitive despite 1–2.8% point lower ASYS detection during CPR than the standard requirement (95%) for non-noisy ECG signals. This is overall a common limitation of other state-of-the-art studies. We consider that the presented deep learning strategy is a basis for improved CPR practices involving both continuous CC and CC with insufflations, associated with minimal CC interruptions for reconfirmation of non-shockable rhythms (minimum hands-off time) and early treatment of fibrillation (minimal pre-shock pauses). 

## Figures and Tables

**Table 1:** Description of the learning and test OHCA databases during CPR, given as the total number of patients and the number of 40 s periods of interest (patients) extracted per rhythm VF, NSR, ONR, and ASYS.

Database	Total Number of Episodes (Patients *)	Number of Episodes (Patients) per Rhythm			
		VF	NSR	ONR	ASYS
Learning	7173 (1504)	409 (172)	175 (82)	1976 (611)	4613 (1092)
Test	6397 (1334)	393 (145)	177 (83)	1848 (544)	3979 (916)
Total	13570 (2838)	802 (317)	352 (165)	3824 (1155)	8592 (2008)

* The total number of patients is less than the sum of all patients per rhythm because one patient intervention might include more than one rhythm type.

**Table 2:** Test performance vs. sHOT estimated for CNN-CPR models with analysis durations of 5 s, 10 s, and 15 s, corresponding to the mean values of the distributions in Figure 9.

	CNN-CPR (5 s)				CNN-CPR (10 s)				CNN-CPR (15 s)			
sHOT	Se	Sp	Sp	Sp	Se	Sp	Sp	Sp	Se	Sp	Sp	Sp
	(VF)	(NSR)	(ONR)	(ASYS)	(VF)	(NSR)	(ONR)	(ASYS)	(VF)	(NSR)	(ONR)	(ASYS)
(s)	(%)	(%)	(%)	(%)	(%)	(%)	(%)	(%)	(%)	(%)	(%)	(%)
0	87.8 *	99.3	95.4	90.6 *	91.4	97.8 *	95.1	88.5 *	92.3	99.2	94.2 *	85.7 *
(0–1]	90.1	99.7	97.7	94.6 *	92.3	99.0	95.9	93.0 *	93.2	100	94.2 *	91.1 *
(1–2]	93.0	99.6	98.5	96.9	94.0	99.4	97.7	96.4	94.8	100	95.2	94.8 *
(2–3]	95.8	99.6	99.3	97.8	96.2	99.6	98.5	97.5	96.0	100	97.6	96.4
(3–4]	95.8	100	99.6	99.0	96.7	100	99.2	98.5	97.1	100	99.1	98.0
(4–5]	96.6	99.9	99.2	99.6	97.8	100	99.1	99.3	97.2	100	99.1	98.8
(5–6]					99.0	100	99.3	99.2	97.7	100	99.0	99.2
(6–7]					98.8	100	99.2	99.4	98.0	100	98.9	99.3
(7–8]					98.8	100	98.8	99.7	98.5	100	99.0	99.7
(8–9]					98.7	100	98.5	99.7	98.7	100	99.1	99.8
(9–10]					98.9	100	97.7	99.6	98.9	100	99.0	99.8
>10									98.9	100	98.7	99.5

* Se, Sp values not satisfying the AHA goals for AED performance in noise-free episodes, Se (VF) > 90%, Sp (ASYS) > 95%, Sp (ONR) > 95%, Sp (NSR) > 99% [47].

## Data Availability

Restrictions apply to the availability of these data. Proprietary data were used, which may be available on reasonable request from the author J-P.D. with the permission of Schiller Médical SAS, Wissembourg, France.
